# PHOrming the inflammasome: phosphorylation is a critical switch in inflammasome signalling

**DOI:** 10.1042/BST20200987

**Published:** 2021-12-02

**Authors:** Chloe M. McKee, Fabian A. Fischer, Jelena S. Bezbradica, Rebecca C. Coll

**Affiliations:** 1 The Wellcome-Wolfson Institute for Experimental Medicine, Queen's University Belfast, Belfast, Antrim, U.K.; 2 Kennedy Institute of Rheumatology, Nuffield Department of Orthopaedics, Rheumatology and Musculoskeletal Sciences, University of Oxford, Oxford, U.K.

**Keywords:** inflammasome, inflammation, NLRP3, phosphorylation/dephosphorylation, post translational modification

## Abstract

Inflammasomes are protein complexes in the innate immune system that regulate the production of pro-inflammatory cytokines and inflammatory cell death. Inflammasome activation and subsequent cell death often occur within minutes to an hour, so the pathway must be dynamically controlled to prevent excessive inflammation and the development of inflammatory diseases. Phosphorylation is a fundamental post-translational modification that allows rapid control over protein function and the phosphorylation of inflammasome proteins has emerged as a key regulatory step in inflammasome activation. Phosphorylation of inflammasome sensor and adapter proteins regulates their inter- and intra-molecular interactions, subcellular localisation, and function. The control of inflammasome phosphorylation may thus provide a new strategy for the development of anti-inflammatory therapeutics. Herein we describe the current knowledge of how phosphorylation operates as a critical switch for inflammasome signalling.

## Introduction

Over the past two decades, cytosolic protein complexes called inflammasomes have emerged as central mediators of inflammation [1,2]. Inflammasomes are formed by sensor proteins, notably including members of the Nod-like receptor family (NLRs), the adapter Apoptosis-associated speck-like protein containing a CARD (ASC) and an inflammatory caspase, caspase-1. The formation of the inflammasome complex provides a platform for the activation of caspase-1 that in turn cleaves the pro-inflammatory cytokines pro-interleukin (IL)-1β and pro-IL-18 into their active forms [1,2]. Caspase-1 also cleaves Gasdermin D (GSDMD), generating an N-terminal fragment which forms pores in the plasma membrane that cause lytic cell death known as pyroptosis [3]. This highly inflammatory signalling process requires multiple levels of regulation to prevent damaging inflammation and the development of inflammatory diseases [1,4]. Inflammasome proteins are regulated transcriptionally (e.g. pro-IL-1β expression induced by Toll-like receptor and cytokine signalling), post-transcriptionally (e.g. microRNA-223 regulates NLRP3), and post-translationally [5,6]. Post-translational modifications (PTMs) including ubiquitination, acetylation, nitrosylation, alkylation, and sumoylation allow fast control of inflammasome function and act as a critical licencing step in inflammasome activation [7,8]. However, phosphorylation is the predominant PTM that has been studied in inflammasome signalling [9]. The reversible transfer of the γ-phosphate of adenosine 5′-triphosphate (ATP) by kinase enzymes to Serine (Ser), Threonine (Thr), or Tyrosine (Tyr) amino acid residues can profoundly affect protein function by regulating protein activity, subcellular localisation, and protein–protein interactions. Phosphorylation is controlled by ∼518 kinases (1.7% of all human genes) and 140 phosphatases that function across many subcellular compartments in human cells [10–13].

## Regulation of inflammasome sensors

Inflammasome sensors are activated either directly by pathogen-associated molecular patterns (PAMPs), danger associated molecular patterns (DAMPs) or indirectly by homeostasis-altering molecular patterns (HAMPs) [14]. Phosphorylation can regulate inflammasome sensors by affecting their inter- and intra-molecular interactions, and their subcellular location. Below we discuss how phosphorylation impacts the most well-studied inflammasome sensors namely, NLRP3, Pyrin, NLRC4, AIM2 and NLRP1.

### NLRP3

**Figure 1.**
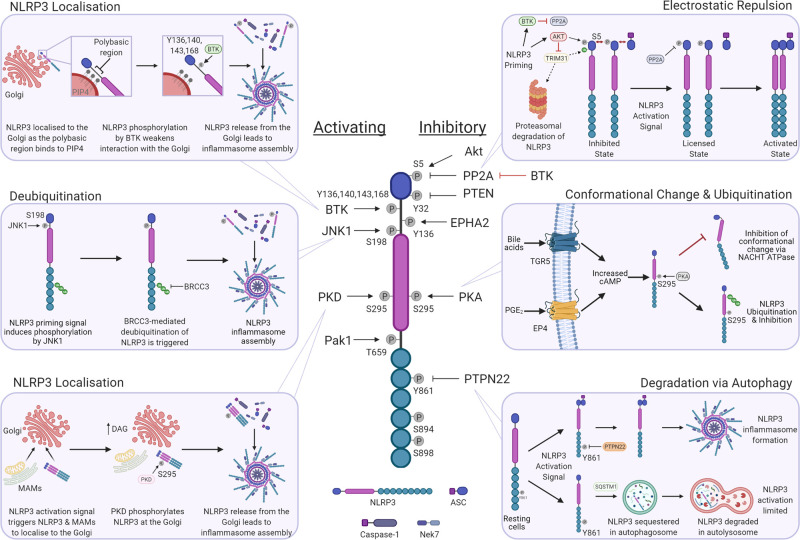
Phosphorylation of NLRP3. NLRP3 inflammasome activation is regulated by phosphorylation, which can be activating (left) or inhibitory (right). The kinases and phosphatases responsible are included where known and the mechanisms of activation/inhibition of some of these modifications are highlighted.

NLRP3 is the most versatile inflammasome sensor in the diversity of stimuli it recognises. It does not directly recognise ligands but detects changes in cellular homeostasis induced by the activity of numerous molecules [15]. This indirect mode of activation may require the high level of phospho-regulation observed in the NLRP3 pathway. We describe NLRP3 phosphorylation with reference to specific residues and their functional effects (Figure 1).

#### Serine 5 (mouse Ser3)

Stutz et al. [16] identified phosphorylation of Ser5 as an inhibitory modification that limits NLRP3 activation. Ser5 is in helix 1 of the pyrin (PYD) domain which forms an interaction interface between the PYDs of NLRP3 and ASC. The negative charge introduced by phosphorylation at Ser5 results in electrostatic repulsion between PYDs, preventing PYD–PYD interactions and thus inhibiting NLRP3 inflammasome formation [16–18]. Recently, AKT (protein kinase B) was identified as a kinase that can mediate phosphorylation of Ser5, while dephosphorylation of Ser5 by protein phosphatase 2 A (PP2A) is essential for NLRP3 activation [16,17]. Intriguingly, phosphorylation of Ser5 plays an additional, contrasting role in NLRP3 regulation by stabilising NLRP3 expression. Phosphorylation of Ser5 prevents TRIM31-mediated ubiquitination of NLRP3, consequently inhibiting proteasome-mediated NLRP3 degradation [17,19]. Thus, Ser5 phosphorylation stabilises NLRP3 expression in response to LPS priming but acts as a brake to limit NLRP3 activation. Ser5 phosphorylation is additionally regulated by the activity of Bruton's tyrosine kinase (BTK) [20]. In LPS-primed macrophages and dendritic cells, NLRP3 is bound to BTK which phosphorylates and inactivates PP2A, preventing Ser5 dephosphorylation. Upon stimulation with an NLRP3 activation signal BTK dissociates from NLRP3 allowing PP2A-mediated dephosphorylation of Ser5 and oligomerisation of NLRP3 [20].

#### Serine 163

Phosphorylation of Ser163 was identified in mass spectrometry data from two separate studies [21,22]. Interestingly, Ser163 is not conserved in mouse or rat NLRP3 but is conserved in primates. However, Ser163Ala mutation does not appear to have a functional effect, as IL-1β processing and interaction with NEK7 were similar to wild-type NLRP3 in reconstitution experiments in HEK293T cells [21,22]. Whether Ser163 could be redundant with other phosphorylation sites, or if a phosphomimetic mutant may affect NLRP3 function remains to be examined.

#### Serine 198 (mouse Ser194)

Ser198 is located between the PYD and NACHT domains of NLRP3. Phosphorylation of Ser198 by c-Jun N-terminal kinase 1 (JNK1) is detected within 15 min of LPS stimulation, demonstrating the rapid nature of transcription independent NLRP3 priming. Signalling from multiple TLRs can induce Ser198 phosphorylation, including TLR1/2, TLR3, TLR5, TLR7, and TLR9. The mutation of Ser198 to a non-phosphorylatable amino acid or the use of JNK inhibitors disrupts homotypic NLRP3 interactions. Ser198 phosphorylation thus regulates NLRP3 oligomerisation and inflammasome assembly [21]. In addition, phosphorylation of Ser198 promotes the deubiquitination of NLRP3 by BRCC3 which is also required for NLRP3 inflammasome formation, highlighting the potential interplay between phosphorylation and other PTMs [21,23]. A recent study using Ser198Ala NLRP3 in a HEK293T reconstitution system did not observe any functional changes in NLRP3 activation [22]. While Ser198Ala has been extensively characterised in macrophages and *in vivo*, these contrasting data highlight the importance of validating observations using phosphorylation mutants in multiple systems [21,22].

#### Serine 295 (mouse Ser291)

Ser295 is in the central NACHT domain and is phosphorylated by protein kinase A (PKA) and PKD [24–26]. PKA is activated by increasing levels of cyclic AMP that can be induced by multiple pathways including signalling induced by bile acids and prostaglandin E2 [24,25]. Mortimer et al. [25] demonstrated that Ser295 phosphorylation inhibits the ATPase activity of NLRP3, which is essential for conformational changes necessary for inflammasome formation. Guo et al. observed that phosphorylation of Ser295 induced ubiquitination of NLRP3. This ubiquitination inhibited NLRP3 via a mechanism that was independent from proteasome or autophagy-mediated degradation but is yet to be fully elucidated [24]. Of potential clinical significance, Cryopyrin Associated Periodic Syndrome (CAPS)-associated mutations in NLRP3 adjacent to Ser295 were observed to have less PKA-mediated phosphorylation and ubiquitination [24,25]. The hyperactive NLRP3-driven inflammation in some CAPS patients may thus be due to a reduced ability of PKA to suppress NLRP3 [24,25]. In a contrasting study, Zhang et al. [26] found that Ser295 phosphorylation by PKD was an activating modification. In response to NLRP3 stimuli, mitochondria-associated ER membranes (MAMs) and NLRP3 are recruited to the Golgi. Enhanced diacylglycerol levels at the Golgi activate local PKD which phosphorylates Ser295 and releases NLRP3 from MAMs allowing ASC recruitment and inflammasome assembly in the cytosol [26]. These results emphasise the importance of NLRP3 localisation to and subsequent dissociation from the Golgi, which is known to be an important event in NLRP3 inflammasome activation [27]. A recent pre-print study by Heiser et al. [28] demonstrated that the PKD inhibitor CRT0066101 could attenuate NLRP3 responses, confirming a requirement for PKD in NLRP3 activation. Further studies are needed to explain the opposing effects of Ser295 phosphorylation by PKA and PKD, but these findings highlight the dynamic spatial and temporal nature of NLRP3 regulation by phosphorylation.

#### Serine 894/898 (mouse Ser891/895)

Ser891 and Ser895 are in the C-terminal leucine-rich repeat (LRR) domain. Wang et al. [29] identified that the phosphorylation of these two serine residues in mouse macrophages allows binding of the E3 ubiquitin ligase TrCP1, which in turn ubiquitinates NLRP3 at Lysine 380 thereby promoting its proteasomal degradation. The binding of TrCP1 can be blocked by the Hippo pathway protein YAP which therefore stabilises NLRP3 [29]. It remains to be elucidated which kinase and phosphatase regulate the phosphorylation status of Ser891 and Ser895, whether they act redundantly, and if this mechanism is conserved in humans.

#### Threonine 659 (mouse Thr657)

A recent study by Dufies et al. [22] observed that Thr659 is a critical site for NLRP3–NEK7 interaction that is regulated by phosphorylation during *Escherichia coli* infection. The binding of NLRP3 and NEK7 is triggered by NLRP3 stimuli and facilitates the formation of bipartite interactions between NLRP3 monomers [30,31]. The *E.coli* virulence factor CNF-1 targets the Rho GTPase Rac2 which triggers p21-activated kinase 1 (Pak1)-mediated phosphorylation of Thr659 thereby inducing NLRP3–NEK7 interactions and inflammasome formation [22]. Whether other stimuli trigger Thr659 phosphorylation is currently unknown.

#### Tyrosine 32 (mouse Tyr30)

Huang et al. [32] identified the lipid and protein phosphatase PTEN as a regulator of NLRP3 inflammasome assembly. They observed that PTEN directly interacts with NLRP3 and dephosphorylates Tyr32 within the PYD domain, facilitating NLRP3–ASC interactions [32]. PTEN is an important tumour suppressor while NLRP3 also contributes to the response of cancer cells to some chemotherapeutic agents [33,34]. Mice with PTEN-deficient myeloid cells exhibit decreased chemotherapy-induced NLRP3 activity in the tumour microenvironment and resistance to Mitoxantrone, suggesting that PTEN-induced NLRP3 activity promotes anti-tumour immunity and tumour suppression [32]. The kinase responsible for Tyr32 phosphorylation is currently unknown.

#### Tyrosine 136 (mouse Tyr132)

NLRP3 contributes to host defence against viruses and a recent study on Reovirus, a double-stranded RNA virus which infects the respiratory tract, identified NLRP3 activation in airway epithelial cells (AECs) [35]. Reovirus infection triggered phosphorylation of NLRP3 Tyr132 mediated by the transmembrane tyrosine kinase receptor EphA2. Tyr132 lies between the PYD and NACHT domains and its phosphorylation appears to limit NLRP3 inflammasome assembly [35]. This negative regulation may prevent excessive inflammasome activation in response to RNA virus infection. However, the phosphorylation of Tyr132 by EphA2 may be specific to AECs as EphA2 is barely detectable in immune cells [35].

#### Tyrosine 136, 140, 143, 168 (mouse Tyr132, 136, 164)

Ito et al. [36] first identified a positive role for BTK in regulating NLRP3. They found that BTK inhibitors such as Ibrutinib specifically blocked NLRP3 activation, while Xid mice (that express functionally inactive BTK) had reduced NLRP3-dependent inflammation *in vivo*. Liu et al. [37] observed similar results with BTK inhibitors and Btk-deficient mice, and importantly observed decreased NLRP3 inflammasome activation in primary myeloid cells from patients with X-linked agammaglobulinemia (XLA) that have mutations in BTK. Both groups observed that BTK could directly interact with NLRP3 and ASC, but whether BTK could directly phosphorylate NLRP3 was unknown [36,37]. A recent study from Bittner et al. [38] identified direct BTK-mediated phosphorylation of NLRP3 and linked this to the subcellular location of NLRP3. A polybasic motif in the linker region between the PYD and NACHT of NLRP3 is critical for binding to phosphatidylinositol-4-phosphate on the trans-Golgi network and subsequent inflammasome activation [27]. Bittner et al. identified four novel phosphorylation targets of BTK, NLRP3 Tyr136, Tyr140, Tyr143 and Tyr168, the first three of which are located within the polybasic region. Stimulation of NLRP3 with nigericin induces BTK-mediated phosphorylation of NLRP3 that weakens the interaction between NLRP3 and the Golgi. NLRP3 is consequently released, allowing NLRP3 oligomerisation and inflammasome formation in the cytosol [38]. However, as discussed above in relation to Ser5, Mao et al. [20] have characterised BTK as a negative regulator of NLRP3. These contradictory findings may be partly attributed to differences in the concentration of LPS used and mouse models employed by the different studies, and these experimental differences are extensively discussed by Mao et al. [20]. It is also possible that BTK is required to regulate distinct stages of NLRP3 activation. Thus, during priming BTK may keep PP2A and NLRP3 activity in check by controlling Ser5, but when an activation signal is sensed and the dispersed TGN forms BTK then phosphorylates NLRP3 at the polybasic motif, to facilitate its release from the TGN. Although most of the current evidence currently suggests BTK activity is required for NLRP3 inflammasome activation, further studies are necessary to clarify the role of BTK in NLRP3 regulation.

#### Tyrosine 861 (mouse Tyr858)

The LRR domain of NLRP3 is not essential for its activation, but phosphorylation of Tyr861 within the LRR has been identified as a negative regulator of NLRP3 [39,40]. Spalinger et al. [40] reported that protein tyrosine phosphatase non-receptor 22 (PTPN22) directly interacts with NLRP3 in an ASC-dependent manner, inducing dephosphorylation of Tyr861. Following NLRP3 activation, phosphorylated NLRP3 interacts with Sequestosome 1 (SQSTM1), a molecule that is involved in targeting substrates to the phagophore. This pathway limits inflammasome activity as only NLRP3 that has been dephosphorylated by PTPN22 will escape recruitment to the autophagosome, thus being protected from degradation and allowing inflammasome assembly [41]. The kinase responsible for Tyr861 phosphorylation is currently unknown, but autophagy also mediates the degradation of inflammasome components including pro-caspase-1, pro-IL-1β, and ubiquitinated NLRP3, highlighting the closely interwoven pathways of inflammasome activation and autophagy [42–44].

### Pyrin

Pyrin is a 95 kDa protein encoded by the MEFV gene. Mutations in MEFV cause inflammatory syndromes including Familial Mediterranean Fever (FMF) and Pyrin-Associated Autoinflammation with Neutrophilic Dermatosis (PAAND) [45–47]. Over 15 years ago it was shown that pyrin signalling is dependent on the phosphorylation state of Ser208, Ser209, and Ser242 [48]. In a yeast 2-hybrid screen in HeLa cells, Jéru et al. [48] found that these phosphorylations enable the 14-3-3 family of scaffolding proteins to bind to pyrin. However, the kinases involved and the significance of pyrin phosphorylation in immune cells was unknown. Further studies revealed that in myeloid cells the GTPase RhoA activates serine-threonine kinases of the PKC superfamily, PKN1 and PKN2 (also termed PRK1/PRK2), to phosphorylate pyrin at Ser208 and Ser242 (Ser205 and Ser241 in mice). Phosphorylation at these sites keeps pyrin inactive in the steady state by allowing its binding to several isoforms of 14-3-3 proteins [49,50]. Three independent groups then showed that pathogens such as *Yersinia pestis* and *Clostridium difficile*, or chemical triggers that inhibit GTPase RhoA, activate the pyrin inflammasome by preventing its phosphorylation and binding to 14-3-3 scaffolds [49–51]. Thus, like NLRP3, pyrin senses the disruption of cellular homeostasis rather than specific ligands.

Unsurprisingly, pathogens have also developed evasion mechanisms that exploit pyrin phosphorylation. For example, the *Yersinia pestis* effector molecule YopM acts as a bridging molecule between pyrin, the host kinases PKN1 and PKN2 and the ribosomal protein S6 kinases 1-3 to facilitate pyrin phosphorylation and increase 14-3-3 binding. This prevents pyrin activity resulting in reduced bacterial clearance [51,52].

PAAND is an autoinflammatory syndrome caused by an Ser242Arg mutation, that prevents Ser242 phosphorylation and the inhibitory interaction of pyrin with 14-3-3 proteins [47]. FMF-associated mutations on the other hand are found in the B30.2/SPRY domain of pyrin where they decrease the threshold for activation, likely by removing the requirement for microtubules in pyrin inflammasome formation [53,54]. FMF mutations on their own do not activate pyrin but combined with either broad-spectrum PKC family inhibitors or with PAAND Ser242Arg mutation (which all block Ser242 phosphorylation) cause excessive auto-activation of pyrin and subsequent inflammation [47,55]. These data suggest at least two critical check-points in pyrin inflammasome activation: one dependent on microtubules, and another on phosphorylation of Ser242. The sensitivity to PKC inhibitors can be used to diagnostically distinguish FMF patient monocytes, which rapidly activate the pyrin inflammasome in response to PKC inhibition [56].

A recent preprint study conducted by Mangan et al. [57] found that stimulation with toxin B from *C. difficile* (TcdB) was also not sufficient to activate the pyrin inflammasome in human macrophages. Although short term LPS priming increased Ser242 phosphorylation which was removed by stimulation with TcdB, this did not, on its own, induce pyrin activation. Prolonged LPS priming was required to increase pyrin expression and activation in response to TcdB [57]. These results further support the hypothesis that in human macrophages Ser242 phosphorylation is not the only checkpoint that prevents pyrin activation.

**Figure 2.**
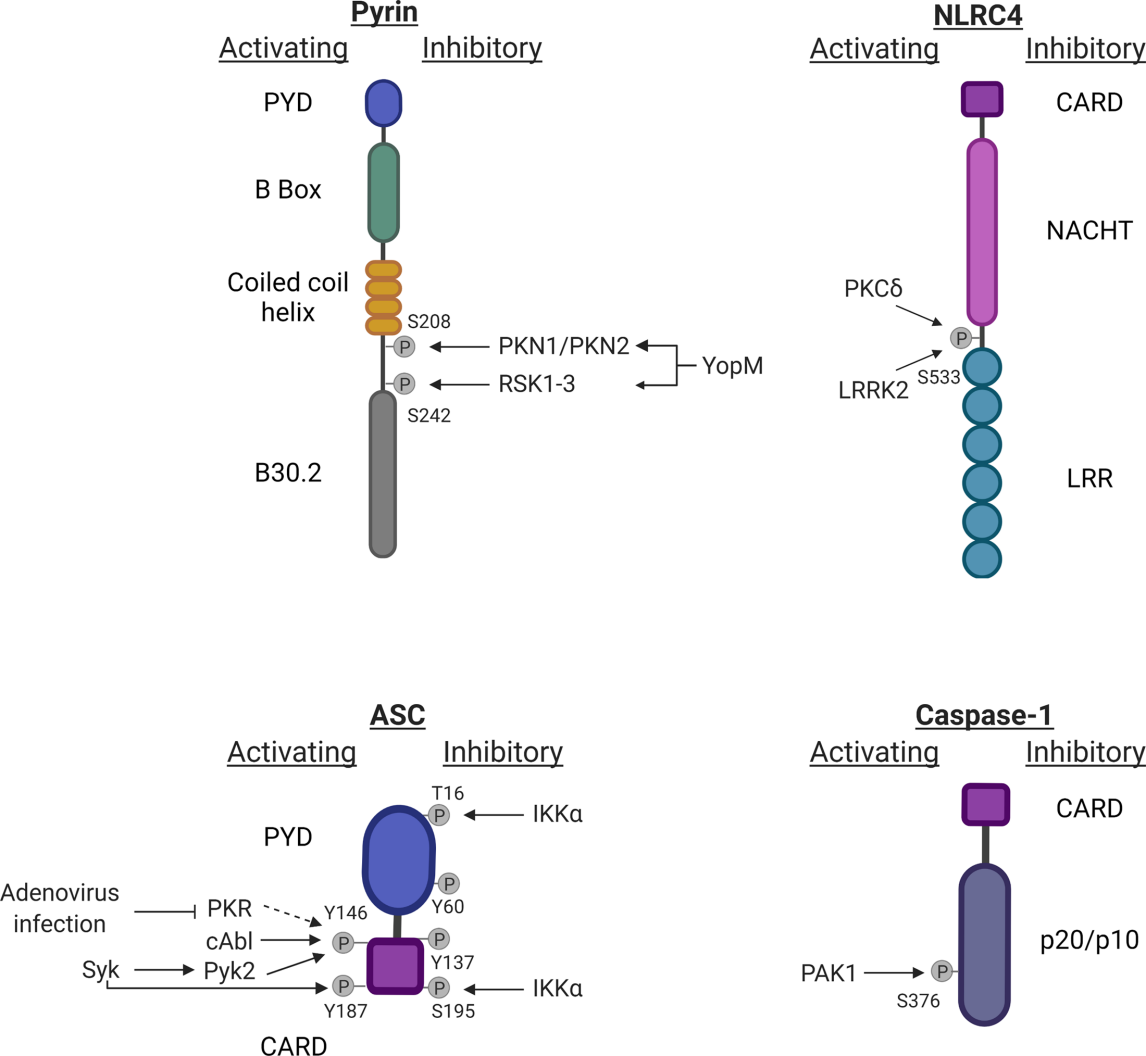
Phosphorylation of inflammasome sensors, ASC, and caspase-1. Phosphorylation of the inflammasome sensors pyrin and NLRC4, ASC and caspase-1 can regulate inflammasome activation. These modifications can be activating (left) or inhibitory (right) and the kinases and phosphatases responsible are included where known.

Interestingly, FMF mutations are not always detrimental. In the Turkish population, they underwent positive selection during periods of the bubonic plague, a deadly disease caused by *Yersinia pestis*. FMF mutations prevent *Yersinia* effector protein YopM binding to the B30.2 domain and subsequent YopM-facilitated Ser208 and Ser242 phosphorylation and pyrin inhibition [52]. Resistance to YopM inhibition allowed FMF carriers to launch pyrin-dependent inflammation that conferred increased fitness against *Yersinia pestis* during bubonic plague [52]. To date no phosphatase has been described to dephosphorylate pyrin, and the exact chronological hierarchy between changes to the phospho-sites and the B30.2 domain is unknown. Pyrin phosphorylation sites and associated regulators are illustrated in Figure 2.

### NLRC4

The NLRC4 inflammasome is a sensor of bacterial invasion. It recognises the presence of flagellin and type III secretion systems by detecting cytosolic rod and needle proteins [58]. Qu et al. [59] first described NLRC4 phosphorylation at Serine 533 by PKCσ. In immortalised mouse macrophages reconstituted with NLRC4 carrying a Ser533Ala mutation, this modification was found to be critical for ASC speck assembly and caspase-1 processing [59]. Further work using an NLRC4 Ser533Ala knock-in mouse model showed that bone marrow-derived macrophages (BMDM) from this mouse did not show a complete reduction in NLRC4 response against *Salmonella* Typhimurium infection, but rather a delayed response relative to wild-type NLRC4 [60]. The leucine-rich repeat kinase (LRRK2) was also shown to phosphorylate Ser 533, raising the question whether its activity is redundant with PKCσ or whether different kinases are activated in distinct cell types and infection models [61].

Interestingly, a separate study proposed that Ser533 phosphorylation can occur independently of NAIP5, the adaptor molecule responsible for flagellin recognition in the NLRC4 inflammasome complex. This study also found that Ser533 phosphorylation can be triggered by a broad range of flagellins, including those that do not lead to NLRC4-dependent IL-1β production, such a *Helicobacter pylori* [62]. These observations led to a model where Ser533 phosphorylation is an essential priming step for NLRC4, although it is not tiggered by direct flagellin recognition and is insufficient to cause NLRC4 activation. A contrastive recent study using an independently generated NLRC4 Ser533Ala knock-in mouse only observed small differences in wild-type versus NLRC4 Ser533Ala BMDM stimulated with cytosolic flagellin or *Salmonella* Typhimurium [63]. Tenthorey et al. used the same mutation and stimulation protocol as Qu et al. thus it remains to be determined what caused the opposing results on the function of Ser533 phosphorylation [60,63]. Potential reasons could include different animal housing conditions that lead to changes in the microbiome, or different cell culture conditions for BMDM e.g. the amount of L929 media used, 5% versus 20% [60,63].

Ser533 is in the NLRC4 HD2 domain that binds to the LRR region and may regulate NLRC4 autoinhibition [64]. Hu et al. [64] suggested that Ser533 phosphorylation strengthens the HD2–LRR interaction, indicating a negative regulatory role for phosphorylated Ser533. To date, no phosphatases have been described that dephosphorylate this site, which would likely be needed to disrupt the HD2–LRR interaction and allow NLRC4 activation. This auto-inhibitory role for phosphorylated Ser533 contradicts the activity-promoting role proposed by others and is derived from the structure of the protein expressed in insect cells. Further studies are needed to determine what role Ser533 phosphorylation plays in different cell types and whether other groups can reconcile the existing data on the Ser533Ala mutant *in vivo*. NLRC4 phosphorylation sites and associated regulators are illustrated in Figure 2.

### AIM2 and NLRP1

AIM2 senses infection and danger by directly detecting the presence of cytosolic double-stranded DNA [65–67]. NLRP1 can be activated indirectly upon cleavage of its N-terminus by pathogen derived proteases such as *Bacillus anthracis* Lethal Factor or enteroviral 3C proteases [68–70]. Recently, it was also shown that NLRP1 can be activated directly by binding long dsRNA generated during Semliki Forest virus infection in epithelial cells [71]. No phosphorylation sites responsible for regulating the activity of either AIM2 or NLRP1 have been characterised, although several putative sites have been identified in high-throughput screens according to PhosphoSitePlus® [72]. Interestingly, AIM2 has inflammasome-independent roles in mouse models of colon cancer and in Experimental Autoimmune Encephalomyelitis (EAE), a mouse model of multiple sclerosis [73–75]. In cancerous colonic epithelial cells AIM2 binds and inactivates the kinase DNA-PK, which is needed for AKT activation and regulates colon cancer progression [73]. In EAE, AIM2 interacts with PP2A to limit AKT activity in T cells [75]. Both studies suggest that AIM2 and AKT can interact and possibly influence each other's activity. However, the canonical AIM2 inflammasome pathway is insensitive to AKT inhibition [17]. Whether AIM2 or NLRP1 are regulated by phosphorylation, and if so, which kinases control them, remains to be discovered.

## Regulation of the inflammasome adapter ASC

The adaptor protein ASC is involved in inflammasome formation for all sensors, although it is not essential for NLRC4 and NLRP1 [2]. ASC is therefore a key target for regulation, and phosphorylation controls ASC localisation and its interaction with other inflammasome complex proteins. ASC phosphorylation sites and associated regulators are illustrated in Figure 2.

### Tyrosine 146 (mouse Tyr144)

Hara et al. [76] showed that Spleen Tyrosine Kinase (Syk) activity is required for phosphorylation of ASC at Tyr144 (human Tyr146). This phosphorylation is crucially needed for ASC speck oligomerisation, although direct phosphorylation could not be shown in an *in vitro* kinase assay [76]. A later study found that Syk additionally phosphorylated ASC at Tyr187. Single mutations of either Tyr146 or Tyr187 did not abrogate ASC speck formation pointing towards a redundant role for these residues [77]. Chung et al. [78] expanded these models and suggested that ASC phosphorylation is indirectly mediated by Syk, as Syk activates the kinase Pyk2 which then phosphorylates Tyr144. Chung et al. also found that the activity of another member of the focal adhesion kinase (FAK) family, FAK was required for IL-1β production. However, FAK could not directly phosphorylate Tyr144 implying a role for FAK at a different site in ASC or at a different step in the pathway [78]. In addition, protein kinase R (PKR) has been described to bind multiple inflammasome sensors, and PKR inhibition decreased all inflammasome activities such caspase-1 cleavage, and IL-1β, and HMGB1 secretion [79]. This conclusion has been challenged in other experimental models suggesting there may be cell type or stimulus specific effects of PKR in inflammasome regulation [80,81]. Darweesh et al. [82] observed that during adenovirus infection PKR is prevented from binding to ASC and that PKR inhibition reduces ASC Tyr146 phosphorylation in THP-1 cells. Whether PKR is a direct Tyr146 kinase or a scaffold for other kinases, and if this interaction is specific to viral infections remains to be answered. More recently, Gavrilin et al. [83] identified cAbl kinase as a regulator ASC phosphorylation at Tyr146 in THP-1 cells. The cAbl-deficient THP-1 cells had reduced, but not absent levels of phosphorylated Tyr146, suggesting that other kinases such as Syk and PKR may act redundantly at this site [83]. Interestingly, cAbl deletion affected only NLRP3, but not other ASC-dependent inflammasomes, but a mechanism remains unclear.

### Tyrosine 60 and 137 (mouse Tyr60, Tyr137 unique to humans)

There are also two possibly inhibitory phosphorylations described for ASC at Tyr60 and Tyr137. Broad inhibition of protein tyrosine phosphatases (PTPases) was found to block NLRP3- and AIM2-mediated IL-1β production that was dependent on these two residues [84]. The kinases and phosphatases that regulate Tyr60 and Tyr137 are currently unknown.

### Mouse serine 16 and 193 (human Thr16 and Ser195)

Phosphorylation also influences the intracellular location of ASC. Martin et al. [85] demonstrated that ASC is retained in the nucleus in the steady state by binding to IKKα via ASC Ser16 and Ser193. During LPS priming, the ASC-IKKα complex is translocated to the perinuclear area, in a process dependent on inhibitory-κB kinase epsilon (IKKε, also known as IKK-i). The inflammasome-activating signal two leads to activation of the phosphatase PP2A which binds to and inactivates IKKα thereby releasing ASC in the cytosol for inflammasome assembly [85]. As described earlier, PP2A also dephosphorylates NLRP3 suggesting that PP2A regulates at least two steps in the NLRP3 pathway [16]. As PP2A knockdown inhibited NLRP3 but not AIM2 responses, it remains to be investigated whether a redundant phosphatase such as PP1 could play an additional role for ASC trafficking upon AIM2 activation [85]. ASC does not contain a nuclear localisation sequence (NLS), and in many cells including BMDMs it is found in the cytosol. This poses the question how and when ASC gets translocated in the nucleus. Hara et al. [76] also reported that ASC is mainly located in or around the nucleus bound to phosphorylated JNK. Contrary to ASC, JNK does contain an NLS, making it a possible interaction and translocation partner.

## Regulation of inflammatory caspases

Caspase-1 is a directly inflammasome-associated caspase that is phosphorylated by the p21-associated kinase PAK1 at Ser376 [86]. In monocytic THP-1 cells stimulated with *Helicobacter pylori* LPS, Ser376 is phosphorylated and leads to caspase-1 activation (Figure 2) [86]. As described earlier PAK1 also regulates NLRP3 at Thr659 during *E. coli* infection, suggesting PAK1 regulates multiple inflammasome proteins during bacterial infection. In contrast with the inflammatory caspases, phosphorylation of the apoptotic caspases-3, -7, -8 and -9 downstream of multiple pro-inflammatory signalling pathways has been well studied. Nearly all these phosphorylations were found to inhibit caspase activity either by blocking interactions between initiation and effector caspases or by blocking substrate binding [87–91]. Interestingly, LPS can induce caspase-8 phosphorylation and inactivation in neutrophils suggesting it plays a dual role in priming inflammasome activation while blocking apoptotic pathways [89]. A second member of the PAK family, PAK2, has been reported to phosphorylate caspase-7 at multiple residues that prevent its activation by caspase-9 [90,91]. PAK1 and PAK2 display 96% sequence homology indicating the possibility of shared substrates. Any potential functional redundancy between PAK1 and PAK2 in phosphorylating caspase-1 or -7 remains to be investigated. Recent research has shown that the apoptotic caspases-3 and -7 inhibit inflammasome-mediated cell death by cleaving GSDMD into an inactive fragment. Caspase-8 on the other hand can promote inflammatory cell death by releasing the active C-terminal domain of GSDMD [3]. Future work may reveal how the phosphorylation state of caspases as well as their interacting kinases and phosphatases control the balance between apoptosis and pyroptosis.

## Regulation of inflammasome substrates

Cleavage of IL-1β, IL-18, and GSDMD represent the final step of inflammasome activation [92]. IL-1β has one described phosphorylation site at Ser134, but its functional significance remains unknown as mutation of Ser134 did not affect the stability or secretion of IL-1β [93]. No direct phosphorylations have been described for IL-18, but both IL-1β and IL-18 are subject to significant regulation as they are transcribed as inactive pro-forms that require enzymatic cleavage for activation. They are also regulated by other PTMs, such as ubiquitylation [93]. GSDMD is the executioner of pyroptosis and to date has not been found to be directly phosphorylated [94]. However, like other inflammasome substrates, it is also regulated via other PTMs [95]. Interestingly, two other gasdermins, GSDMA and GSDME, were shown to be phosphorylated at Thr6 and Thr8, respectively [96,97]. These phosphorylations block their pore forming capability and inhibit pyroptosis [97]. GSDMD does not carry a Threonine residue within the first 20 amino acids, but a Serine residue at position 3 could be a potential target for a similar mechanism. Future studies will likely uncover more phospho-regulated sites in gasdermins and the phosphatases involved in removing their inhibitory phosphorylations upon inflammasome activation.

## Conclusions

Phosphorylation and kinase/phosphatase signalling pathways control many cellular processes and are particularly prominent in immunity. For example, cytokine receptor signal transduction pathways rely on Jak tyrosine kinases to activate Stat transcription factors [98].The inflammasome pathway is not a classical signalling cascade involving membrane receptors, downstream kinase(s), and activation of transcription factors, but it is now apparent that phosphorylation also plays a critical role in regulating inflammasome activity. The phosphorylation of inflammasome sensors and the adapter ASC control the protein–protein interactions necessary to form the inflammasome complex. Recent research has highlighted how the subcellular localisation of inflammasome proteins is essential for complex formation and is also regulated by phosphorylation. For example, NLRP3 localisation to the *trans*-Golgi network appears to be regulated by PKD and BTK [26,38]. While our knowledge of inflammasome pathway phosphorylation has expanded, it is difficult to reconcile the numerous phosphorylation and dephosphorylation events that have been reported. How inflammasome phosphorylation occurs temporally, spatially, and alongside other PTMs such as ubiquitination is not understood. Whether some phosphorylations are cell type and stimulus specific is also in many cases unclear. The challenges of studying inflammasome phosphorylation include the lack of well characterised phospho-specific antibodies, differences in mouse and human cell models, and the translation of *in vitro* identified phospho-sites to *in vivo* disease relevance (e.g. NLRC4 Ser533) [99,100]. Despite these obstacles there are already reports using specific kinase inhibitors to modulate inflammasomes *in vivo*. For example, the BTK inhibitor Ibrutinib has been shown to reduce NLRP3 activation in mouse models of ischemic brain injury [36] and high-fat diet-induced chronic metabolic inflammation [36,101]. These results suggest that targeting phosphorylation events in the inflammasome pathway is a promising therapeutic approach.

## Perspectives

- The importance of the field: Inflammasome activation and subsequent cell death often occurs within minutes to an hour, so the pathway must be dynamically controlled to prevent excessive inflammation. Phosphorylation is a fundamental PTM modification that allows rapid control over protein function.
- Summary of current thinking: Phosphorylation regulates the subcellular localisation, protein–protein interactions, and functions of inflammasome sensors and adapters and is an important regulatory checkpoint in inflammasome activity.
- Future directions: A deeper understanding of inflammasome phosphorylation, cell type specificity, and *in vivo* disease relevance, will undoubtably provide opportunities to develop novel therapies for inflammasome-mediated diseases.

## Open Access Statement

Open access for this article was enabled by the participation of Queen's University Belfast in an all-inclusive Read & Publish pilot with Portland Press and the Biochemical Society under a transformative agreement with JISC.

## Abbreviations

AECs: airway epithelial cells
BMDM: bone marrow-derived macrophages
BTK: Bruton's tyrosine kinase
CAPS: Cryopyrin Associated Periodic Syndrome
EAE: Experimental Autoimmune Encephalomyelitis
FAK: focal adhesion kinase
FMF: familial mediterranean fever
GSDMD: Caspase-1 also cleaves Gasdermin D
IL: interleukin
LRR: leucine-rich repeat
MAMs: mitochondria-associated ER membranes
NLS: nuclear localisation sequence
PAAND: Pyrin-Associated Autoinflammation with Neutrophilic Dermatosis
PKA: protein kinase A
PKR: protein kinase R
PP2A: protein phosphatase 2 A
PTMs: Post-translational modifications
PTPN22: protein tyrosine phosphatase non-receptor 22
PYD: pyrin
